# Cancer Education Status in China (2013–2022)

**DOI:** 10.1007/s13187-024-02487-w

**Published:** 2024-08-23

**Authors:** Li Yitian

**Affiliations:** Department of Hygiene, Public Health College, Jining Medical University, Jining, Shandong China

**Keywords:** Cancer control; Cancer education; China; Rural–urban disparity

## Abstract

The incidence and mortality rates of cancer in China have an increasing trend, with a remarkable rise in the number of new cases and deaths. Despite this, cancer profile and regional distribution remained relatively stable. China realized a series of initiatives and issued strategic documents to improve cancer education. These include the establishment of a three-tier cancer prevention program and the fulfillment of various guidelines and plans, including the Healthy China Action—Cancer control Implementation Plan (2019–2022). This comprehensive review describes the status of cancer education in China from 2013 to 2022 discussing the role of different cancer education places and that of educators. It also highlights the use of innovative educational methods and educational evaluations, which are effective in improving patient outcomes and satisfaction. Although the Chinese government has taken many measures to improve cancer education in China, several issues remain unresolved. Challenges such as the wide spectrum of tumors, the aging population, and the huge urban–rural disparities require further investment from Chinese government. In addition, cancer control in China started relatively late and lacks the support of specific legislation to control it. It is therefore necessary to increase the investment in cancer education, especially in rural areas and the legislation in areas related to cancer control should be improved to increase the accessibility and quality of education on tumor prevention and treatment.

## Introduction

The onset of malignant tumors is a multistep development process involving multiple factors, with complex pathogenic mechanisms and rapid development, making prevention and control difficult. Previous reports suggest that factors such as tobacco use, unhealthy diet, lack of exercise, overweight or obesity, occupational hazards, chronic irritation, and environmental pollution increase the risk of cancer incidence [1–4]. An estimated 19.3 million cases of cancer occurred globally in 2020, with 18.1 million cases excluding non-melanoma skin cancer, and nearly 10.0 million cancer-related deaths, or 9.9 million excluding non-melanoma skin cancer [5]. Projected estimates for the year 2040 suggest a significant increase in the global cancer, with a projection of 28.4 million new cases, marking a 47% increase over 2020 figures [5]. This increase is expected to be more pronounced in transitioning economies, where the increase is expected to range from 64 to 95%, compared to that of the transitioned economies, which are predicted to increase from 32 to 56% [6]. This disparity is attributed to demographic changes, although the impact may be further amplified by the increasing prevalence of risk factors related to globalization and economic expansion.

Cancer incidence and mortality rates in China increased between 2013 and 2022 [7, 8], and their profiles, as well as the characteristics of the regional distribution, remained virtually unchanged. The comparison of the year 2022 with 2013 revealed that the estimated number of new malignant tumor cases in China increased from 3.682 million cases (2.0486 million in males; 1.6334 million in females) to 4.8747 million cases (2.5339 million in males; 2.2908 million in females). Specifically, the number of new malignant tumor cases in urban areas increased from 1.1486 million to 2.9039 million, while it increased from 1.5334 million to 1.9208 million in rural areas. In terms of mortality, the estimated number of malignant tumor deaths in China grew from 2.2293 million cases (1.4062 million in males; 0.8231 million in females) to 2.5742 million cases (1.6293 million in males; 0.9449 million in females). The correlation of the population data of China in 2013 and 2022 revealed that the crude incidence rate of malignant tumors in China increased from 27 per 100,000 to 35 per 100,000. Similarly, the crude mortality rate for malignant tumors increased from 163 per 100,000 to 182 per 100,000; the top five cancers in China in 2013 were lung cancer, female breast cancer, gastric cancer, liver cancer, and colorectal cancer. By 2022, the top five cancers by incidence were lung cancer, colorectal cancer, thyroid cancer, liver cancer, and female breast cancer. The increasing incidence of thyroid cancer, especially papillary thyroid carcinoma, is mainly due to the widespread and sophisticated use of ultrasonography together with the increased use of various diagnostic imaging techniques. However, the mortality rate for the top five causes of cancer death did not change, still being lung cancer, liver cancer, gastric cancer, colorectal cancer, and esophageal cancer. In terms of regional distribution, the incidence rate of malignant tumors among urban residents is generally higher than that among rural residents. Nevertheless, the mortality rate from tumors among urban residents is significantly lower than that among rural residents.

China is actively addressing the growing burden of cancer in its population [9]. According to the Statistical Bulletin on the National Economic and Social Development of China in 2013 and the Statistical Bulletin on the National Economic and Social Development of China in 2022, the total population of China increased from 1.367 billion to 1.412 billion between 2013 and 2022, representing a growth of 3.3%, and the population aged 60 and over grew from 202 to 280 million, an increase of 38.6%. Within this demographic, the number of individuals aged 65 and older increased from 132 to 210 million, representing a significant increase of 59.1%. The increase of old people in the population, coupled with the prolonged survival of patients with chronic diseases, is increasingly highlighting the burden of cancer among the elderly. Additionally, the rapid development of urbanization and industrialization, together with the prevalence of unhealthy lifestyle behaviors and environmental risk factors [10–12], poses substantial challenges to the efforts of controlling cancer in China.

This review summarized some of the cancer education studies and outcomes and listed some education methods and contents. The search terms for this review were education (or health education) on cancer control (or prevention; or prevention and control) in China. The references are mainly derived from Internet databases such as China National Knowledge Infrastructure (CNKI), PubMed, and Web of Science. The search was restricted to studies published in Chinese or English from 2013 to 2022, and some government documents were also included.

## Current Status of *Cancer* Education in China

China started a series of actions and issued relevant documents to ensure the improvement of cancer education and the accuracy of the related educational content. China launched a three-level cancer prevention program that includes the control of risk factors, promotion of public health, vaccination campaigns, and workplace cancer prevention. Subsequent guidelines have been issued [13], including the National Cancer Control Planning Outline (1986–2000), China Cancer Control Planning Outline (2004–2010), China Cancer Control Three-Year Action Plan (2015–2017), China Chronic Disease Prevention and Control Mid and Long-Term Plan (2017–2025), Healthy China Action—Cancer Control Implementation Plan (2019–2022), and Healthy China Action (2019–2030). These guiding documents underscore the importance of establishing a comprehensive cancer education system and providing the residents with the correct knowledge on cancer education. Specifically, the Healthy China Action (2019–2030) [14] directly targets to improve the awareness rate of the main knowledge on cancer control among residents to over 70% by 2022 and over 80% by 2030 by providing detailed explanations on cancer prevention, healthy lifestyles, reduction of cancer-related infections, and cancer screening. These documents not only provide guidance on cancer control but also enrich the content of cancer education [44–46].

This review presents the status of cancer education from aspects such as educational sites, educators, target audience, educational content, educational methods, educational outcomes, and evaluation in China from 2013 to 2022.

### Education Places and Educational Objects

The analysis of literature indicates that the groups involved in cancer education mainly include the family, schools, hospitals, workplaces, and communities [15–17]. Among them, different places such as urban communities, rural areas, primary and secondary school campuses, factories, and hospitals have already carried out health education for cancer control.

The current health education primarily targets cancer patients their families, some students, and workers. Among them, cancer patients have a relatively low level of knowledge about cancer control, which calls for emphasis and improvement of health education on the control of cancer [18]. Medical staff is also one of the targets of cancer education. Some studies revealed that more than 60% of medical staff and more than 80% of patients believe that hospitals should perform cancer education, thus improving its level on the medical staff, to facilitate the development of personalized health education programs for the prevention and control of oncology [19].

### Educators

Cancer education for cancer patients in China was originally provided mainly by nurses during ward rounds and bedside nursing care, but in recent years, the role of physicians and other staff has increased. The health education pathway model uses time as the horizontal axis and clinical care programs as the vertical axis to develop a feasible and scientifically standardized program that is rigorously applied [20]. This model involves the joint participation of physicians and nurses, ensuring professional division of labor and comprehensive cooperation, which shortens the process from illness to rehabilitation [21], for example, peer support education and self-help group activities to improve the psychosocial adaptation of postoperative colorectal cancer patients [22] and health education-related quality control circles on functional recovery of breast cancer patients [23]. Medical and health professionals are essential in cancer control, and the effectiveness of cancer education carried out by medical personnel is relatively good [24].

### Educational Contents

The content of cancer education in China mainly involves three aspects: knowledge related to cancer control, attitude related to cancer control, and behavior related to cancer control. The first aspect mainly includes information on the possibility of preventing cancer, the basic knowledge of secondary cancer prevention (early detection, early diagnosis, and early treatment), common cancer risk factors, and early symptoms of common cancers [25–27]. The National Bureau of China’s Disease Control and Prevention compiled the Main Information and Key Points of Cancer Control, which was promoted during the National Cancer Control Publicity Week in 2020 [28]. This document focused on the fundamental information of knowledge on cancer control, providing it in an easily accessible way to the public. Attitudes related to cancer control include willingness to participate in cancer education, willingness to receive vaccinations, and willingness to change unhealthy behaviors [29]. Behaviors related to cancer control include performing regular physical exercise, monitoring body weight, quitting smoking, having regular check-ups, and seeking timely medical care. Health education activities performed in hospitals mainly involve knowledge on diseases, treatments, nursing care, medications, and diet [30, 31].

### *Educational Methods (*Table 1*)*

**Table 1:** New educational methods in Chinese cancer education (2013–2022)

Name	Design	Realization	Evaluation of the methodologies and outcomes*
The Parent–Child Keyword method [32] cancer education	1. This method identifies a subheading called the Parent Keyword based on the fundamental cancer education content 2. Based on the content of the Parent Keyword, one or more fundamental phrases are summarized, referred to as the Child Keywords 3. The nurses adjust the number of Child Keywords according to Parent Keyword	1. Inclusion criteria: (1) patients diagnosed by endoscopy who meet the indications for surgical treatment by endoscopic mucosal dissection (ESD); (2) patients who signed the surgery notification letter 2. Exclusion criteria: (1) use of anticoagulant drugs before enrollment; (2) patients with combined heart, liver, lung and other organ failure 3. 90 patients at the early stage and with precancerous lesions of the digestive tract admitted to ESD from January 2018 to December 2018 were selected for the study	1. Increase of the score of self-designed disease knowledge scale from 21.22 ± 4.25 to 34.20 ± 5.23 2. Increase of the score of self-care ability scale from 87.82 ± 5.23 to 132.10 ± 8.23 3. Decrease of the patients’ time of postoperative recovery from 2.96 ± 0.89 to 1.25 ± 0.36 days 4. Increase of the patient satisfaction rate from 35 to 43%
The flowchart method [33] cancer education	A cancer education manual according to the process including admission, preoperative period, morning of surgery, postoperative period, recovery period and discharge from hospital	1. Seventy patients, 42 males and 28 females, aged 29 to 68 years, with an average age of 53.15 ± 3.33 years, who underwent tongue cancer surgery in the ENT department of the hospital from January 2013 to December 2014 2. Thirty-five patients operated from January 2013 to December 2013 were set as the control group, and 35 patients operated from January 2014 to December 2014 were set as the experimental group 3. Comparison of age, gender, and education level between the two groups that showed no statistically significant difference	1. The scores of the self-designed illness cognition scale in the treatment group were higher than those of the control group (50.63 ± 2.07 vs. 34.94 ± 4.08) 2. The tongue activity was better in the treatment group than that of the control group 3. The language clarity was better in the treatment group (86.63 ± 3.15 vs. 65.23 ± 3.52) 4. The satisfaction rate was higher in the treatment group (35/35 vs. 31/35)
The mind map method [34] cancer education	The mind map uses different colors to represent different topics and the number of identical topics is 7 ± 2. The mind map method cancer education is delivered in detail according to the process of “identification of problems-introduction of knowledge-repetition and consolidation-summarization.”	1. Inclusion criteria: (1) lung cancer confirmed by clinical diagnosis; patients who received peripherally inserted central venous catheter (PICC); (2) TNM staging I–III; no other prior treatment or intervention; (3) good compliance 2. Exclusion criteria: (1) diseases of other organs such as heart and liver; (2) abnormal coagulation function; pleural effusion ≥ 20 mL; (3) Tumor metastasis to pulmonary hilum or large blood vessels; (4) cognitive dysfunction, blurred consciousness; (5) incomplete clinical data 3. A total of 60 lung cancer patients who were subjected to chemotherapy were enrolled: 30 patients from June–December 2019 as the control group and 30 patients from January–June 2020 as the treatment group	1. The scores Self-Rating Anxiety Scale (SAS; 52.27 ± 4.48 vs. 65.46 ± 4.38) and Self-Rating Depression Scale (SDS; 50.64 ± 4.35 vs. 64.55 ± 4.72) were lower than those in the control group 2. The self-management ability scores were higher than those in the control group, such as taking medication as prescribed (18.07 ± 0.38 vs. 15.72 ± 2.61 points) and regular review (18.52 ± 0.17 vs. 15.39 ± 1.74 points) 3. The survival status scores were higher than those of the control group, such as pain relief (9.39 0.09 vs. 7.28 1.27 points) and action capability (9.47 0.28 vs. 7.08 1.45) 4. The complication occurrence rate (10.00%; 3/30) was lower than that in the control group (33.33%; 10/30)
The pathogenic factors based interventions [35] cancer education	This method focuses on patients’ attention on the pathogenic factors of breast cancer. It guides patients on how to cooperate with the nursing care for the treatment and how to take care of themselves	1. Inclusion criteria: (1) married female patients; (2) first course of chemotherapy after radical mastectomy for breast cancer; (3) aged 25–55 years; (4) conscious, without cognitive impairment, and able to express their own feelings; (5) no history of psychiatric illness and other serious physical diseases; (6) willing to participate in this study 2. Exclusion criteria: (1) patients with distant tumor metastasis; (2) patients with severe mental illness 3. A total of 100 breast cancer patients admitted in 2015 were included: 50 cases as the treatment group and 50 cases as the control group	1. The passing rate of shoulder joint function exercise in the treatment group was higher than that in the control group (88.0% vs. 62.0%) 2. The rate of postoperative complications in the treatment group was lower than that in the control group (16.0% vs. 40.0%) 3. The recovery rate for the activities of Daily Living (ADLs) in treatment group was higher than that in the control group (76.0% vs. 52.0%) 4. The satisfaction rate was higher in the treatment group than that in the control group (100.0% vs. 82.0%)
The combination of PBL and CBS [36] cancer education	The combination of Problem-Based Learning (PBL) and Case-Based Study (CBS) in cancer education	1. Inclusion criteria: (1) patients with transcatheter arterial chemoembolization (TACE) for hepatocellular carcinoma; (2) patients without severe mental and language disorders; (3) informed consent was read and signed before the study 2. Exclusion criteria: (1) patients with other serious organ diseases; (2) patients who do not actively cooperate with medical care; (3) patients who do not participate in the process of health education activities for 2 or more times 3. Hundred patients eligible for enrollment from October 2017–February 2018 were enrolled in the control group; 100 patients eligible from March–August 2018 were enrolled in the treatment group	1) The scores of Hamilton Anxiety Scale were lower in the treatment group (2.72 ± 1.38 vs. 10.36 ± 5.61) than those in the control group 2) The scores of the self-designed patient knowledge mastery questionnaire were higher in the treatment group (19.96 ± 0.20 vs. 12.93 ± 3.14) than those in the control group
The static and dynamic overlay education model [46]	1. The static education includes the production of breast cancer health education coloring pages, a patient version of the breast cancer health education pathway, and a personalized discharge rehabilitation plan booklet 2. The dynamic education includes face-to-face education, peer education, encounter groups about breast cancer education, multimedia playback and WeChat short-video education	1. The inclusion criteria were the following: (1) age 25–70 years, (2) initial diagnosis of unilateral breast cancer, (3) undergoing modified radical mastectomy for breast cancer, no preoperative coexisting limb dysfunction, (3) no coexisting serious diseases in other systems, and (4) signed informed consent by the patients 2. Uncooperative persons and coexisting psycho-cognitive communication disorders were excluded 3. Hundred-six cases who underwent modified radical mastectomy for breast cancer in June 2016 (53 cases) and May 2017 (53 cases)	1. Increase of the scores of the self-designed Breast Cancer Disease-Related Knowledge and Skill Acquisition Rating Scale (reliability of 0.821; validity of 0.83) from 52.94 ± 3.48 to 91.89 ± 3.29 2. The completion rate of the post-intervention rehabilitation behavioral schedule was 96.23% 3. Increase of the scores of the quality life questionnaires (QLQ) from 52.25 ± 3.61 to 75.47 ± 3.17
The bundle cancer education [47]	1. They review the current state of cancer education in the hospital and analyze the cancer education literature to develop the bundled cancer education program based on their inquiry 2. The bundle health education is conducted on the basis of the evidence 3. Its content includes: a personalized health education pathway, some personalized cancer education content, and cancer education videos with content updated in real time	1. Inclusion criteria: (1) diagnosed and surgically treated skull base tumor, (2) age above 14 years, and (3) have the ability to understand correctly 2. Exclusion criteria: impaired consciousness not enabling to accept health education 3. Patients with skull base tumors from January-December 2015 were studied. A total of 1051 cases were enrolled, 534 males and 517 females	1. Increase of the passing rate of the self-designed cancer education checklist from 83.1 to 97.3% 2. Decrease of the hospitalization days from 16.06 ± 4.37 to 12.71 ± 5.12 days 3. Increase of the scores of the patients’ satisfaction with cancer education surveys from 8.71 ± 0.39 to 9.58 ± 0.23 points
The enhanced cancer education on the four tracks of the main axis [48]	The main axis is the effective completion of health education information 1. The individualized education track focuses on providing individualized education manuals based on patient characteristics and needs 2. The systematic education track provides targeted health education before, during and after chemotherapy 3. The hierarchical division complementary education track focuses on building a hierarchy of cancer education providers who can compensate for each other's gaps and make continuous improvements 4. The distance education track is mainly used to push health education information through multiple channels and answer patients’ questions through QQ groups, WeChat groups and telephones	1. Inclusion criteria: (1) diagnosis of lung cancer, (2) who received chemotherapeutic intervention, (3) no distal migration of the lesion, (4) Kahn’s score (KPS) not less than 70, (5) signed informed consent 2. Exclusion criteria: (1) coexisting renal, hepatic, and cardiac failure, (2) expected survival of less than 6 months, and (3) presence of impaired consciousness 3. 80 lung cancer patients who received chemotherapy in the hospital from December 2016 to June 2018 were selected as study subjects: 47 males and 33 females	1. Significant increase of the scores of self-designed cancer knowledge scale (reliability of 0.809; validity of 0.814) such as medication knowledge (from 18.19 ± 1.54 to 23.48 ± 1.25) 2. Increase in the number of chemotherapy accidents from 8 to 2 cases 3. Significant increase of the scores of the self-developed cancer education satisfaction scale (reliability of 0.805; validity of 0.807) such as educational content (from 9.44 ± 0.62 to 6.90 ± 1.17 points)

*All the outcomes were statistically significant (*p* < 0.05)

The Parent–Child Keyword method [32] formulates a cancer education sheet for precautions in the endoscopic submucosal dissection (ESD) based on the literature and clinical practice. The method summarizes preoperative and postoperative precautions and provides a detailed summary of cancer education content. First, this method identified a subheading called the Parent Keyword based on the fundamental cancer education content. Then, one or more fundamental phrases are summarized based on the content of the Parent Keyword, referred to as the Child Keywords. The Parent Keywords belong to the outline of cancer education, while the Child Keywords are supplements to the description of the Parent Keywords (Table 2). The nurses adjust the number of Child Keywords according to Parent Keyword. The Parent–Child Keyword cancer education improves patients’ knowledge of diseases and self-care ability in the treatment of early cancer and precancerous lesions in the digestive tract by ESD. It is also beneficial for postoperative recovery and in the improvement of the satisfaction rate with medical services.

**Table 2:** Examples of some Parent–Child Keywords for endoscopic mucosal stripping

Time	Parent Keywords	Child Keywords
Preoperative period	Gastrointestinal preparation	Fasting and abstaining from drinking for 8 h
	Skin preparation	Nasal cavity cleaning
	Personal hygiene	Bathing and changing clothes, trimming nails
	Informed consent	Signo of anesthesia and surgery consent
Postoperative period	Posture intervention	Semi-reclining position or supine position
	Pain care	Music intervention, analgesic pump
	Psychological care	Emotional relaxation, confidence enhancement
	Comfort care	Playing music or videos for the patient

The Flowchart method [33] consists of a cancer education manual according to the processes of admission, preoperative period, morning of surgery, postoperative period, recovery period, and discharge from the hospital. This method uses a combination of centralized lectures and bedside guidance for tongue cancer patients. It helps in the improvement of the effect of patient rehabilitation training, promote the recovery of tongue and voice functions, and improve the quality of life. It also increases the patients’ disease knowledge and the satisfaction rate.

The mind map method [34] also improves the effect of health education in lung cancer patients. This method uses different colors to represent different topics, and the number of identical topics was 7 ± 2. The cancer education knowledge is carefully explained to the patients in accordance with the cancer education process called “identifying problems-introducing knowledge-repeating and consolidating-summarizing.” The mind map is then provided to both the patient and their family members to guide the patient in forming standardized self-management behaviors. The complication occurrence rate and SAS and SDS scores of the patients in the treatment group were significantly lower than those in the control group. Furthermore, the scores of self-management ability and the survival status were significantly higher in the treatment group than those in the control group.

He Ting [35] and others applied the pathogenic factors based interventions to improve the satisfaction of breast cancer patients. Traditional health education is performed with a result-oriented method, while this method starts with the pathogenic factors, such as the causes of breast cancer. The method focuses on patients’ attention to dietary choices, adherence to treatment, regular follow-up, rehabilitation exercises, emotional and sexual management, and other health behaviors. It guides patients on how to collaborate with nursing care for treatment and how to take care of themselves and helped patients to better understand the purpose and importance of cancer education. The passing rate of shoulder joint function exercise (88.0% vs. 62.0%), the recovery of the ADLs (76.0% vs. 52.0%) and the satisfaction rate were higher, and the rate of postoperative complications (100.0% vs, 82.0%) was lower in the treatment group than those in the control group.

Li Ying [36] and others introduced the combination of PBL and CBS into cancer education, achieving remarkable results. The current conventional method easily leads to anxiety in patients during the perioperative period of TACE [37], and it also reduces patients’ quality of life and the efficiency of medical and nursing work. The most popular PBL [38–40] promotes self-learning motivation and improves problem-solving skills, and it is widely applied in the field of medicine. In addition, CBS is based on patients who successfully solved practical problems, using typical clinical cases to analyze the basic knowledge, mechanisms, and principles of patients one by one, deepening the understanding, as well as cultivating and improving the ability of patients to use the learned knowledge to solve practical problems [41–44]. This method significantly reduces the anxiety level of patients (2.72 ± 1.38 vs. 10.36 ± 5.61 points) and improves their knowledge related to cancer education (19.96 ± 0.20 vs. 12.93 ± 3.14).

The dynamic and static overlay education [46] is divided into two parts: static education and dynamic education. Static education includes personalized coloring pages, a rehabilitation plan booklet and other handouts. The dynamic education includes face-to-face education, peer education, encounter groups on breast cancer education, multimedia playback and WeChat short-video education. The dynamic and static overlay education improves the disease control rehabilitation knowledge and skill acquisition of breast cancer patients (from 52.94 ± 3.48 to 91.89 ± 3.29 points), as well as rehabilitation behavioral adherence (96.23%), and quality of life (from 52.25 ± 3.61 to 75.47 ± 3.17 points).

Wang Binlin and others [47] improved the cancer education bundle in patients with skull base tumors. They reviewed the current state of cancer education in the hospital and analyzed the cancer education literature to develop a cancer education bundle program based on the patients’ request. The bundle health education is performed according to the evidence. Its content includes a personalized health education pathway, some personalized cancer education content, and cancer education videos with the content updated in real time. The cancer education bundle increases patients’ knowledge of cancer education (percentage of qualified people from 83.1 to 97.3%), reduces the length of hospitalization (from 16.06 ± 4.37 to 12.71 ± 5.12 points), and increases patients’ satisfaction with the health education (from 8.71 ± 0.39 to 9.58 ± 0.23 points).

Chen Jianying [48] used the enhanced cancer education which has the four tracks of the main axis to improve the satisfaction of patients under lung cancer chemotherapy with cancer education. The main axis represents the effective completion of health education information. The four tracks include the individualized education track (content based on personal needs), the systematic education track (targeted before, during and after chemotherapy), the hierarchical division complementary education track (stratified management for educators), and the distance education track (through multiple channels like QQ, WeChat, and telephones). The knowledge of medication (from 18.19 ± 1.54 to 23.48 ± 1.25), adverse reactions (from 18.04 ± 1.18 to 23.65 ± 1.21), drug extravasation management (from 18.31 ± 1.24 to 23.46 ± 1.17), and chemotherapy self-care (from 17.90 ± 1.16 to 23.50 ± 1.20) are the significantly improved aspects of the enhanced cancer education. In addition, the number of chemotherapy accidents decreased from 8 to 2. Finally, patients were more satisfied with the education system (from 9.38 ± 0.64 to 7.29 ± 1.07), the education pathway (from 9.38 ± 0.64 to 6.25 ± 1.14), the education method (from 9.50 ± 0.62 to 6.85 ± 1.25), and the educational content (from 9.44 ± 0.62 to 6.90 ± 1.17) after the enhanced cancer education.

### Educational Outcomes

#### Effectiveness Evaluation According to Patient Feedback

Some oncology education studies focus on the effectiveness of the oncology education delivery according to the feedback from oncology patients. Zhang Ting [49] and colleagues used narrative nursing intervention to guide patients to express themselves, provide answers, analyze key problems and resolve them accordingly. Xia Minjing [50] and colleagues used a clock-hour representation intervention, adhering to the basic logic from “identifying errors” to “solving problems,” which significantly corrected the biases and knowledge deficiencies of cancer patients. As regards the Knowledge-Attitude-Practice (KAP) Model, the study of Wu Xiaoyan and others [31] used it on cancer patients, revealing that the KAP cancer education intervention improves the compliance and satisfaction of cancer patients.

#### Questionnaire-Based Effectiveness Evaluation for Residents

Many studies in China target the general population and use questionnaires to statistically analyze data from the baseline and follow-up surveys to obtain the effects of cancer education activities. For instance, Peng Wei et al. [51] and Wu Ni et al. [52] performed studies on health intervention for cancer prevention among a large number of residents and the survey results before and after the intervention were compared. The results indicated that the residents’ awareness of cancer-related knowledge significantly improved after the intervention compared to their knowledge before the intervention. According to the requirements of the Healthy China Action-Cancer Control Action Implementation Program (2023–2030) [53], the main knowledge of cancer control will reach more than 80% by 2030. Therefore, cancer education research in China began to see the emergence of studies around 2020 through questionnaire targeting the awareness rate of the fundamental knowledge on cancer control [16].

## Discussion

All the above pieces of evidence highlighted that China carried out a series of measures from 2013 to 2022 to improve the level of cancer education. Initially, China increased the number of educators in cancer control and improved the professional level of these educators. Subsequently, the integration of cancer control knowledge into fundamental information and key knowledge points led to the systematization of cancer education content and its practicality was emphasized. Concurrently, various forms of education performed by hospitals at all levels and public organizations stimulated the initiative of learners and educators, thereby improving the effectiveness of education. Lastly, hospitals at all levels and public organizations ensured the quality of cancer education in China through different evaluation methods and subjects. Although China achieved some success in cancer education, there are still some issues that need to be improved.

Firstly, the spectrum of cancer incidence in China is broad, with significant differences between urban and rural areas, affecting the effectiveness of cancer control in China. The composition and ranking of cancer incidence and mortality spectrum in China show a coexistence with the cancer spectrum of developed countries. The incidence of the upper digestive tract cancer, such as esophageal, gastric, and liver cancer, which are prevalent in the former, remains high, while lung, colorectal, and breast cancer, which are common in developed countries, are also rapidly increasing in China [54]. The difficulty of cancer control work is greatly increased if the significant disparities between urban and rural areas and the uneven regional distribution are added to the above problems. The Healthy China Action-Cancer control Action Implementation Plan (2023–2030) proposes to strengthen the promotion of cancer control among rural residents. Indeed, rural residents have a higher mortality rate despite the relatively lower incidence rate as revealed by the regional distribution characteristics of mortality rates from malignant tumors in China in 2013 and 2022. This indicates the existence of a certain gap in cancer education among rural residents in China compared to urban residents at the current stage.

Furthermore, cancer education in China started relatively late mostly remaining at the policy document stage and lacking corresponding legal construction compared to other East Asian countries (such as Japan). Japan established the National Cancer Center in 1962 and promoted the Cancer Control Act in 2006. This legislation not only provides knowledge and content related to cancer education but also clarifies the main body of the propaganda responsibility [55]. For example, the act explicitly states: “Citizens should be committed to acquiring the correct knowledge about cancer, including the impact of smoking, diet, exercise, and other living habits on health, as well as infectious diseases that may lead to cancer….” The act also stipulates: “Medical insurance undertaking parties should cooperate with the national and local public entities in the dissemination and popularization of the knowledge about cancer prevention and cancer screening (including necessary countermeasures based on the results) in accordance with the law; … Doctors and related medical personnel should cooperate with the cancer measures taken by the national and local public entities, and contribute to the prevention of cancer, ….” Moreover, the incidence and mortality rates of cancer continue to rise as the aging population in China gradually increases. Therefore, it is of the utmost importance that the Chinese government legislates cancer control measures as early as possible and incorporates cancer education into relevant laws and regulations. Furthermore, investment in cancer education should be increased for residents in rural areas by expanding the scope of publicity and improving the understanding and mastery of cancer prevention knowledge among rural residents.
